# Deciphering Epithelial–Mesenchymal Transition Regulatory Networks in Cancer through Computational Approaches

**DOI:** 10.3389/fonc.2017.00162

**Published:** 2017-08-03

**Authors:** Gerhard A. Burger, Erik H. J. Danen, Joost B. Beltman

**Affiliations:** ^1^Drug Discovery and Safety, Leiden Academic Centre for Drug Research, Leiden University, Leiden, Netherlands

**Keywords:** epithelial–mesenchymal transition, computational modeling, cancer progression, cell migration, stemness, cell metabolism

## Abstract

Epithelial–mesenchymal transition (EMT), the process by which epithelial cells can convert into motile mesenchymal cells, plays an important role in development and wound healing but is also involved in cancer progression. It is increasingly recognized that EMT is a dynamic process involving multiple intermediate or “hybrid” phenotypes rather than an “all-or-none” process. However, the role of EMT in various cancer hallmarks, including metastasis, is debated. Given the complexity of EMT regulation, computational modeling has proven to be an invaluable tool for cancer research, i.e., to resolve apparent conflicts in experimental data and to guide experiments by generating testable hypotheses. In this review, we provide an overview of computational modeling efforts that have been applied to regulation of EMT in the context of cancer progression and its associated tumor characteristics. Moreover, we identify possibilities to bridge different modeling approaches and point out outstanding questions in which computational modeling can contribute to advance our understanding of pathological EMT.

## Introduction

Epithelial–mesenchymal transition (EMT) is the transition from an epithelial to a mesenchymal phenotype of a cell. During this transition, cells lose their epithelial properties, such as strong cell–cell adhesion, and gain mesenchymal properties, such as spindle-like morphology and enhanced migratory capacity. Together with its reverse process, mesenchymal–epithelial transition (MET), EMT plays an important role in development (type 1 EMT), wound healing and fibrosis (type 2 EMT), and cancer (type 3 EMT) (1).

Because of the increased migratory and invasive capacity of cells with a mesenchymal phenotype, it was thought that EMT at the tumor site and subsequent MET at the metastatic site were required for metastasis (2). Nevertheless, despite the observation of EMT *in vitro*, lack of evidence for EMT *in vivo* caused skepticism about its proposed role in tumor progression (3). Recently, evidence for *in vivo* EMT has been provided by isolation of tumor cells that have undergone EMT (4) and intravital microscopy of such cells in epithelial breast tumors (5). However, the importance of EMT in tumor progression remains a source of debate, as other studies have shown that inhibition of EMT, either by deletion of SNAIL1 (SNAI1) or TWIST1 (6), key EMT transcription factors (EMT-TFs), or overexpression of miR-200 (7), a microRNA that represses EMT, did not affect metastasis in, respectively, pancreatic and lung cancer. Rather, these studies show that inhibition of EMT and induction of MET (re)sensitized cells to chemotherapy, suggesting that EMT contributes to chemoresistance rather than to metastasis (6, 7). Interestingly, a recent study shows that deletion of the EMT-TF ZEB1 in the same pancreatic cancer model does affect metastasis (8). This finding points to the existence of complementary subprograms of EMT driving cancer cell dissemination, i.e., individual EMT-TFs have distinct effects (8, 9).

Whereas the recent findings by Krebs et al. (8) partly resolve the controversy around the contribution of EMT to metastasis, another confounding factor is the existence of plasticity-dependent and plasticity-independent metastatic pathways (10). Yet another factor that plays a role is the frequent assumption that EMT is an “all-or-none” process, in which a cell is either fully epithelial or fully mesenchymal. In contrast to this notion, recent studies have revealed a large flexibility in the EMT process, leading to the recognition of intermediate or “hybrid” phenotypes, in which cells show both epithelial and mesenchymal properties (11, 12). Cells with such hybrid phenotypes have the ability to migrate while remaining attached to other tumor cells, potentially dragging along epithelial tumor cells that cannot migrate on their own. Indeed, genes associated with a mesenchymal phenotype were found to be upregulated in circulating tumor cell (CTC) clusters detected in the blood of breast cancer patients (13), yet the tight adhesion of cells in a cluster suggests they are not fully mesenchymal. Moreover, clusters of melanoma cells can intravasate into blood vessels (14) and clusters of CTCs in breast and prostate cancer patients have a much higher (up to 50-fold) metastatic potential compared to single CTCs (15). Hence, partial EMT rather than complete EMT might be associated with metastasis.

Apart from the immense progress in our understanding of EMT owing to experimental studies, in recent years also computational and mathematical modeling approaches have made a significant contribution to this understanding (16, 17). Here, we review such modeling approaches on (partial) EMT and how they have helped to elucidate regulation and implications of EMT in tumor progression.

## Unraveling the EMT Regulatory Network

Epithelial–mesenchymal transition is regulated by a complex network of interconnected pathways such as the transforming growth factor-β (TGFβ), epidermal growth factor (EGF), insulin-like growth factor, Wnt, Hedgehog, and Notch pathway (18). Of these pathways, the TGFβ pathway is studied the most extensively in the context of EMT. TGFβ is dysregulated in many types of cancer and acts both as a tumor suppressor at early stages of tumor development (by inhibiting proliferation and inducing apoptosis) and as a tumor promoter at late stages (by inducing EMT or by suppressing immune responses against the tumor) (19). As a result of this multifunctional, context dependent [e.g., influenced by tumor extracellular matrix (ECM) rigidity (20)] behavior, there has been considerable interest in understanding the TGFβ pathway mechanistically, and various models of the canonical (Smad-dependent) pathway [reviewed in Ref. (21)] and non-canonical (Smad-independent) pathway (22) have been developed.

Eventually, to induce EMT the signals from the various EMT-activating pathways converge on a network of EMT-TFs that repress epithelial characteristics, such as E-cadherin expression [the loss of which is an important hallmark of EMT (23)], and induce mesenchymal characteristics, such as Vimentin expression. Thus, TFs that support epithelial characteristics are inactivated (24) and those that support mesenchymal characteristics are activated. In this section, we will discuss the modeling efforts that researchers have undertaken to unravel the EMT regulatory network, starting with an EMT “core” regulatory network and working our way up to systems-level approaches.

### Core Regulatory Network

**Figure 1:**
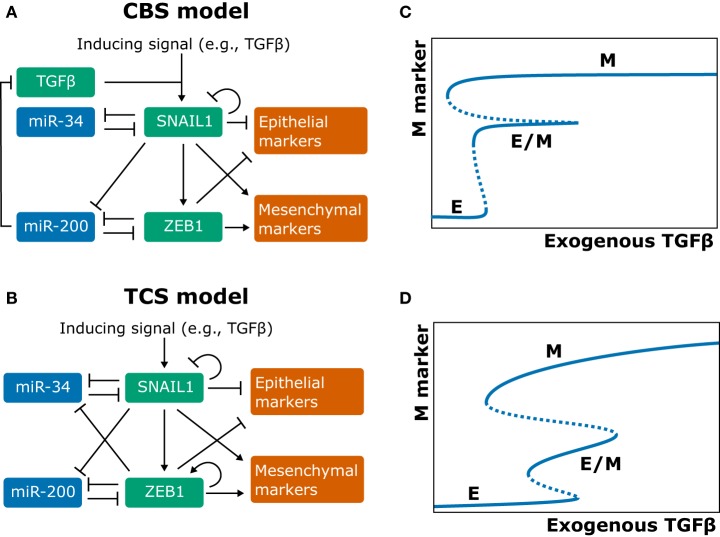
Models of the epithelial–mesenchymal transition core regulatory network and their behavior. **(A,B)** Network graphs of the cascading bistable switches (CBS) model by Tian et al. (25) and Zhang et al. (26) **(A)** and of the ternary chimera switch (TCS) model by Lu et al. (27) **(B)**. **(C,D)** Bifurcation diagrams corresponding to the CBS model **(C)** and TCS model **(D)** [**(B,D)** based on Ref. (27); **(A,C)** based on Ref. (26)].

Obvious candidates for inclusion in the EMT core regulatory network are EMT-TFs such as ZEB1/2, SNAIL, SLUG (SNAI2), TWIST1, and PRRX1 (11, 18). Two independently developed ordinary differential equation (ODE) models describe a part of this core regulatory network by combining two double-negative feedback loops: one between SNAIL1 and miR-34 and one between ZEB1 and miR-200 (Figures 1A,B). These models were termed the cascading bistable switches (CBS) model [(25); later revised by Zhang et al. (26)], and the ternary chimera switch (TCS) model (27). Both models confirm key experimental findings on EMT: first, the occurrence of three stable equilibria representing the epithelial, mesenchymal, and intermediate phenotype (referred to as E state, M state, and E/M state, respectively; see Figures 1C,D), thus allowing for a multistep EMT process. Second, the conditional irreversibility of a complete EMT due to permanent entrapment in the mesenchymal phenotype (26, 27).

The key differences between the competing models are (a) that the CBS model includes an autocrine TGFβ/ZEB/miR-200 feedback loop and (b) that the TCS model includes self-activation of ZEB1 (Figures 1A,B). There is experimental evidence both for the autocrine feedback loop (28) and for a ZEB1 positive-feedback loop *via* splicing factor ESRP1 and stem cell marker CD44 (29, 30). The two model assumptions result in qualitatively different model predictions. First, although both models predict that a complete EMT occurs in two steps, they predict different roles for the SNAIL1/miR-34 and ZEB1/miR-200 modules. Specifically, in the CBS model, the SNAIL1/miR-34 module causes the transition from the epithelial (E) to the hybrid (E/M) state when the inducing signals are weak, whereas for strong signals, the ZEB1/miR-200 module causes the subsequent transition from the E/M to the mesenchymal (M) state (25, 26). In the TCS model, the SNAIL1/miR-34 module acts as a noise-buffering integrator (i.e., it makes the system less sensitive to noise in the inducing signal), and the ZEB1/miR-200 module acts as a ternary switch causing all of the E, E/M, and M states (27). Second, the models predict different mechanisms for the conditional irreversibility of a complete EMT. In the CBS model, the irreversibility of the transition from the E/M to the M state is caused by the autocrine TGFβ/ZEB/miR-200 feedback loop (Figure 1B), which is well established (28) yet not included in the TCS model (26). In the TCS model, the existence of such irreversibility depends on the strength of the inhibition of miR-34 by ZEB1 (27).

Zhang et al. (26) supported their CBS model with data from the human breast epithelial cell line MCF10A. mRNA and protein measurements of SNAIL1, ZEB1, E-cadherin, and Vimentin showed that SNAIL1 abundance is low in the E state and high in the E/M and M state, whereas ZEB1 abundance is high only for the M state. These results are consistent with the CBS model but not with the TCS model because the latter predicts intermediate ZEB1 levels in the hybrid state. Additional flow cytometry analysis indeed failed to show intermediate levels of ZEB1 for cells in the E and E/M states (26). However, since phenotypic heterogeneity can distort population-based measurements, it is hard to distinguish between the two core regulatory models based on the experimental evidence currently available. One way to resolve this is by the acquisition of single-cell, time-course measurements. This could be achieved through time-lapse imaging of reporter cell lines exposed to various stimuli, thus providing detailed insight into the dynamics of EMT regulation.

### Extending the Core Regulatory Network

The core regulatory network models discussed above include only a limited number of regulatory components and even leave out well-established EMT-TFs. For example, PRRX1 cooperates with TWIST1 to induce EMT in a SNAIL-independent manner (31), and it is unclear how PRRX1 and TWIST1 connect to the proposed core regulatory network centered around SNAIL and ZEB. These network elements are thought to have different roles, i.e., PRRX1 and TWIST are strong mesenchymal inducers and weak epithelial repressors, whereas SNAIL and ZEB are strong epithelial repressors and weak mesenchymal inducers (11). Another omission is that of EMT-TF paralogs, which can induce different EMT programs. For example, SNAIL and its paralog SLUG (SNAI2) both have a mutually inhibiting loop with miR-200 (32) and can bind to the ZEB1 promoter; however, ZEB1 expression is controlled by SNAIL and not by SLUG (33). Additionally, TWIST2 expression, and not TWIST1 expression, leads to upregulation of AKR1B1 that promotes basal-like breast cancer progression. AKR1B1 also activates NF-κB which in turn upregulates TWIST2, constituting an EMT-inducing positive-feedback loop (34). By contrast, TWIST1 needs SLUG mediation to induce EMT (35), further illustrating the complexity of EMT-TF interplay, and the need to include this in regulatory models.

Although the abovementioned core regulatory network models have provided insight into EMT regulation, their continued value depends on their ability to explain and predict the effect of newly added regulators. One class of such regulators has been termed “phenotypic stability factors” (PSFs) (36) because they can stabilize the cells in a particular (e.g., a hybrid) phenotype. These PSFs can be, but are not necessarily TFs. In this section, we will discuss extensions of the core regulatory network models with the PSFs OVOL, Grainyhead-like transcription factor 2 (GRHL2), and miR-145.

#### OVOL

**Figure 2:**
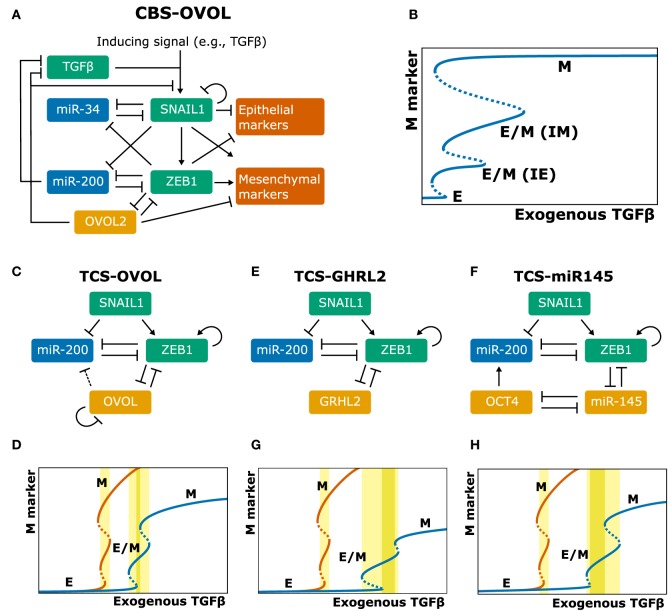
Extensions of the core regulatory models and their behavior. **(A–H)** Model schemes **(A,C,E,F)** and corresponding bifurcation diagrams **(B,D,G,H)** of various extensions of the ternary chimera switch (TCS) and cascading bistable switches (CBS) models: the CBS-OVOL model **(A,B)**, the TCS-OVOL model [**(C,D)**; dashed line in panel **(C)** indicates inhibition of miR-200 by OVOL which occurs in prostate cancer but not in breast cancer], the TCS-Grainyhead-like transcription factor 2 model **(E,G)**, and the TCS-miR145 model **(F,H)**. Bifurcation diagrams of the unmodified TCS model are shown in orange and of modified TCS models in blue. TGFβ ranges are highlighted for which there exists a stable hybrid E/M state (light yellow) and for which this is the only stable state (dark yellow). Note that in the extensions of the TCS model, this model is first simplified by considering SNAIL as input, which can be done because the tristability in that model is fully determined by the miR-200/ZEB module [**(A,B)** based on Ref. (39); **(C–H)** based on Ref. (43, 44)].

OVOL, a TF that regulates embryogenesis, has been reported as a master PSF since it suppresses several known EMT-TFs (36) and can drive MET (37). Moreover, its absence changes TGFβ from growth suppressor to EMT inducer (38). To elucidate the dynamics of OVOL in regulating EMT, Hong et al. (39) extended the CBS model with OVOL (Figure 2A). Because OVOL inhibits TGFβ (38), Hong and coworkers add an additional autocrine TGFβ/ZEB/OVOL feedback loop, strengthening the autocrine TGFβ signaling already present in the CBS model. An interesting prediction from this extended model is the existence of two intermediate phenotypes (Figure 2B). These phenotypes could correspond to the “intermediate epithelial” and “intermediate mesenchymal” phenotypes identified in experiments by Huang et al. (40) and are consistent with a generalized view of EMT as a continuum where cells transition sequentially through multiple intermediate states (11, 41). Hong and coworkers speculate that the MCF10A cell line [used by Zhang et al. (26)] may be “intermediate epithelial” rather than epithelial. This would be consistent with the collective migration displayed by MCF10A cells, a feature associated with an intermediate phenotype (42). Moreover, because the cells hardly express mesenchymal markers, they are more likely “intermediate epithelial” than “intermediate mesenchymal” (39). Overexpressing OVOL in MCF10A cells indeed induced an even more epithelial state, in which E-cadherin expression was similar to the well-characterized epithelial MCF7 cell line (39). Another prediction by Hong et al.’s extended CBS model is that OVOL can induce a complete MET irrespective of high TGFβ levels. Finally, to determine the importance of the three mutual inhibition loops in the system (miR-34/SNAIL, miR-200/ZEB, and OVOL/ZEB), Hong et al. varied the strength of these inhibitions. Although in their model all three loops enable and stabilize the intermediate phenotypes, the OVOL/ZEB loop provides the most pronounced stability, consistent with OVOLs proposed role as “master PSF” (36, 39).

The role of OVOL was also investigated by Jia et al. (43) who extended the TCS model with OVOL and applied this to prostate and breast cancer (Figure 2C). This extended model predicted that the presence of OVOL has various effects on the EMT dynamics (Figure 2D): first, a much higher inducing signal is required to initiate and complete EMT, which is due to the inhibition of ZEB1 by OVOL. Second, the range of the inducing signal for which the hybrid E/M phenotype exists is larger. Third, the hybrid E/M phenotype becomes the only possible phenotype for a certain range of model parameters (43). In contrast to the CBS-OVOL model, OVOL could not always induce a complete MET in the TCS-OVOL model, which is likely caused by the absence of TGFβ inhibition by OVOL. Nevertheless, these findings together do confirm the suggested role of OVOL as “critical molecular brake on EMT” (38).

#### GRHL2 and miR-145

Jolly and coworkers also studied the role of two other proposed PSFs in extended TCS models: GRHL2, a well-known regulator of morphogenesis, and miR-145 (44). These factors couple to the miR-200/ZEB loop (Figures 2E,F) and the models predict they affect the EMT process in a way similar to OVOL (Figures 2G,H). Confirming these model predictions, they showed that knockdown of GRHL2 in the lung adenocarcinoma cell line H1975, a stable hybrid E/M cell line, led to complete EMT. Additionally, analysis of the NCI-60 panel revealed that both OVOL and GRHL2 positively correlated with CDH3 (P-Cadherin), a proposed marker of the hybrid E/M phenotype (45). High levels of OVOL, GRHL2, and CHD3 predict a poor prognosis in patient data across multiple tissue types, emphasizing the aggressiveness of the hybrid E/M phenotype (44).

Although most studies investigating EMT and MET focus on carcinomas that are of epithelial origin, there is also evidence of an “MET-like phenomenon” in sarcomas that arise from mesenchymal cells. MET in sarcomas likely involves the same factors as those controlling EMT in carcinomas (46). To test whether PSFs can also drive MET in sarcomas, Somarelli et al. investigated the effect of GRHL2 in RD (rhabdomyosarcoma) and 143B (osteosarcoma) cells. Unexpectedly, GRHL2 overexpression did not have an effect on ZEB1 or E-cadherin levels in these cells. By contrast, miR-200 transfection led to a significant increase in E-cadherin, but without any effect on cell morphology. However, the combination of GRHL2 overexpression and miR-200 transfection had a synergistic effect on E-cadherin and led to a morphological change consistent with MET. Modification of the TCS model such that ZEB1 inhibited GRHL2 activity yielded model results consistent with the experimental data. This model-predicted inhibition of GRHL2 activity by ZEB1 could be caused by the ZEB1 cofactor BRG1: knockdown of BRG1 while GRHL2 was expressed led to a significant increase in E-cadherin (46).

### Systems-Level Modeling of EMT

Although ODE models such as the core regulatory models presented above help in the quantitative understanding of the network, they require detailed knowledge of which regulators are connected and of the kinetic parameters that govern those interactions. This is problematic for system-level models of EMT that contain a large number of regulators implicated in EMT, including the connection to upstream and downstream signaling events. To deal with these issues, researchers have employed several computational approaches: first, by studying classes of ODE models rather than single ODE models. Second, by studying ODE models with very different parameter combinations yet with similar dynamic behavior. Third, by studying Boolean models which need no parameter estimates. Below we discuss how these approaches have been applied to EMT.

#### Classes of ODE Models

The ERK and Wnt pathways induce EMT *via* activation of SNAIL and SLUG and are thought to be highly connected *via* coupled feedback loops (CFLs) (47). To investigate the functional roles in regulating EMT, Shin et al. (47) combined established, basic ODE models for the ERK and Wnt pathway using six established CFLs as connections between these pathway models. These connections can be turned ON (included) and OFF (excluded), which resulted in 2^6^ = 64 different ODE models (for example, in model F1 F2 F4, CFLs 1, 2, and 4 are ON and CFLs 3, 5, and 6 are OFF). Since CFL F6 had only a minor effect on the response of the system, it was excluded from the analysis, so only 32 models were considered further. The behavior of these models was greatly affected by the exact CFL combination employed, and this shows that combination of a limited number of components can encode “biological specificity” (47), similar to Hanahan and Weinberg’s notion of signaling pathways as “electronic integrated circuits” (48). Simulations with these models predicted an important role for Raf kinase inhibiting protein (RKIP) in shaping the dynamics of ERK activity and E-cadherin expression in response to activation of ERK and/or Wnt signaling. This result may explain why RKIP expression is inversely related to metastasis in many cancers (47).

The view of gene regulatory networks as “integrated circuits” is further illustrated by identification of basic building blocks with specific functional roles, the so-called “network motifs” (49). The two-component miRNA-TF modules of the EMT core regulatory network discussed earlier represent examples of such network motifs and have been studied in-depth to determine their functional role (50, 51). Recently, this approach of determining the functional role of a circuit from its topology has been applied to larger regulatory networks (52). This new computational method of random circuit perturbation suggested that the topology rather than detailed parameter knowledge of a network determines its dynamics. By deriving a 22-component EMT network from literature and interaction databases, their approach predicted four possible network states: epithelial, intermediate epithelial, intermediate mesenchymal, and mesenchymal. Additionally, their analysis suggested that SNAIL and SLUG expression is high in the intermediate epithelial state, whereas ZEB expression is high in both intermediate and full mesenchymal states (52). However, the most probable function of a circuit topology may not be its true function.

#### Parameter Combinations in ODE Models

Another approach to deal with unknown parameters in ODE models of gene regulatory networks is to exploit the “sloppiness” of a system. Sloppiness refers to the observation that predictions from multi-parameter models often mainly depend on a few combinations of key parameters (53). In fact, consideration of a “core regulatory network” for EMT presupposes such sloppiness in EMT regulation. Recently, Gould et al. applied the “sloppy” Pareto optimal ensemble technique (POET) to study TGFβ-induced EMT in the presence and absence of vascular endothelial growth factor A (VEGF-A) (54). Their literature-derived EMT network consists of 97 nodes and 169 edges and has 251 unknown model parameters. Forty-one data sets from DLD1 colon carcinoma, MDCKII, and A375 melanoma cells were used to estimate these parameters. Using these data sets to fit a single model would result in a highly uncertain model, especially because the data sets are obtained from different cell lines. Instead, POETs estimate parameters by generating an ensemble (i.e., population) of probable signaling models that can then together be used to predict the system’s behavior [for details, see Ref. (55)].

It has been suggested that such an ensemble can be used to describe heterogeneous populations (55), i.e., by considering that the variability in the behavior of individual cells stems from parameter variability as estimated within the ensemble. Gould et al. tested this hypothesis by measuring the response of E-cadherin and vimentin to stimulation with TGFβ and/or VEGF-A within their model ensemble (approximately 1,400 models). After TGFβ stimulation, the majority (>80%) of the models showed the expected switch from the epithelial to the mesenchymal phenotype. Surprisingly, concurrent VEGF-A and TGFβ stimulation resulted in a large subset of models achieving a hybrid phenotype with simultaneous E-cadherin and vimentin expression. This subset of models displayed upregulated NFATc activity and inhibition of NFATc in these models “restored” full EMT. Experimental stimulation with VEGF-A and TGFβ in MCF10A and DLD1 cell lines confirmed this prediction, i.e., the absence of an NFATc inhibitor led to high levels of both E-cadherin and vimentin (hybrid phenotype), and its presence led to a full EMT (54).

#### Boolean Models

**Figure 3:**
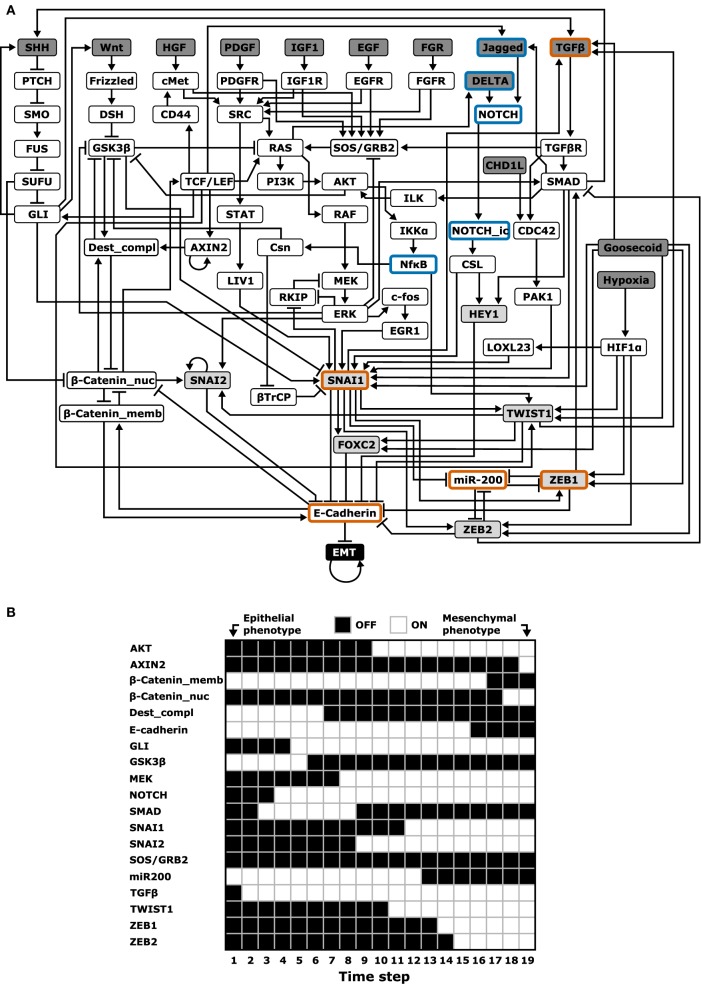
Boolean modeling of epithelial–mesenchymal transition (EMT). **(A)** Boolean model with 70 nodes and 135 edges by Steinway et al. (57). The following network elements are highlighted: upstream regulators (dark gray), EMT transcription factors (light gray), and elements included in the cascading bistable switches and ternary chimera switch core regulatory models (orange border) or their extensions (blue border) [based on Ref. (57)]. **(B)** One of the probable transition paths from the epithelial to mesenchymal phenotype in a reduced, but functionally similar EMT model [cf. Figure 5A in Ref. (57)].

A completely parameter-free approach is Boolean modeling. In Boolean models, genes or other regulators are either ON or OFF, and Boolean models aim to give a *qualitative* description of the system (56). One such Boolean model of EMT was developed by Steinway et al. (57). Based on an extensive literature study, they constructed a TGFβ-driven EMT network in the context of hepatocellular carcinoma (HCC) with 70 nodes and 135 edges (Figure 3A). This model was able to describe the known TGFβ-induced EMT processes (Figure 3B): TGFβ activates the canonical (SMAD) and non-canonical MAPK and AKT pathways, leading to induction of EMT-TFs and ultimately to E-cadherin loss (which is how the occurrence of EMT was defined in this model). Additionally, this model predicted that continued activation of TGFβ activates both the Wnt and Sonic hedgehog (SHH) pathways. This prediction was confirmed in the murine epithelial HCC P2E cell line and in the human epithelial-like HCC cell lines Huh7 and PLC/PRF/5: upon exposure to TGFβ, GLI2 mRNA (indicative of SHH signaling) and AXIN2 protein levels (indicative of Wnt signaling) were both increased to similar levels as in related mesenchymal cell lines (57).

In a follow-up study, Steinway et al. used their model to predict how knocking out various genes would inhibit TGFβ-induced EMT (27, 58). For single-gene knockouts, only manipulation of direct E-cadherin repressors suppressed EMT. For double knockouts involving non-E-cadherin repressors, six two-node combinations fully suppressed EMT (i.e., EMT was OFF in all simulations) and three combinations partly suppressed EMT (i.e., EMT was still ON in some simulations). Interestingly, these nine two-node combinations all involved SMAD as one of the nodes. An experimental test of this prediction by monitoring E-cadherin expression and migration behavior upon siRNA knockdown of these combinations in the human HCC cell line Huh7 revealed that almost all predicted combinations indeed inhibited EMT.

Given the important role of SMAD, Steinway et al. studied the attractor landscape of the EMT network after *in silico* SMAD inhibition. Interestingly, in addition to the mesenchymal steady state observed in the unperturbed model, they found two new stable states: an intermediate epithelial and an intermediate mesenchymal state. The intermediate epithelial state still had epithelial properties such as E-cadherin expression, membrane-localized β-catenin, and inactive SHH, AKT, and Wnt signaling, but also included mesenchymal features such as activated MEK, ERK, and SNAIL. The intermediate mesenchymal state shared this mix of epithelial and mesenchymal features, yet E-cadherin was not expressed, and there was no membrane localized β-catenin.

Steady-state analysis of other single node perturbations of the EMT network revealed a “putative spectrum” of EMT phenotypes. One such perturbation is knockout of ZEB, which induced hybrid steady states (58). Comparing this finding to the earlier discussed core regulatory network ODE models (TCS and CBS) at first sight suggests that this is only consistent with the CBS model because the TCS model predicts an epithelial phenotype upon ZEB knockdown. However, it is an unresolved question how the hybrid phenotypes in Boolean models relate to those in ODE models because in the latter case three different levels of, e.g., E-cadherin can be achieved (low, intermediate, and high). Moreover, the TCS model predicts a hybrid phenotype with intermediate ZEB expression (27). Such intermediate states can thus by definition not be achieved with a two-state Boolean model. An interesting question is therefore whether the use of many-state logic models (59) or qualitative ODE models (60) can reconcile the findings of these models. Another unresolved question concerns the robustness of the results with respect to the EMT regulators included in Steinway’s model. Although the number of regulators substantially exceeds that of the core regulatory network presented earlier, the Boolean model neither includes the identified PSFs OVOL and GRHL2 nor several miRNA families (e.g., miR34) which have also been implicated in EMT (61). Finally, since the model includes multiple signaling pathways, it would be interesting to test if the model can also reproduce EMT induction by other pathways, such as the EGFR pathway (62).

## EMT in Relation to Other Tumor Characteristics

**Figure 4:**
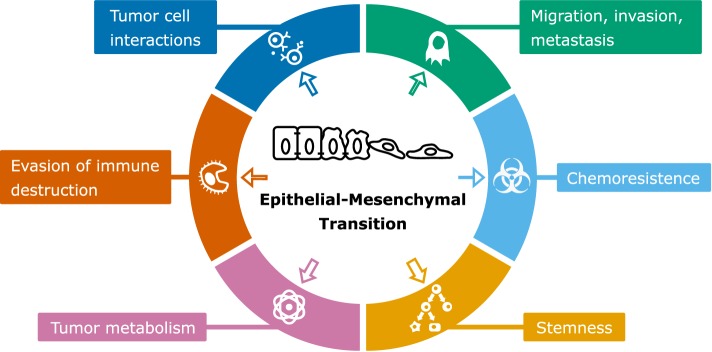
Scheme of epithelial–mesenchymal transition (EMT) and associated cancer characteristics. The size of the arrows indicates the amount of modeling studies focused on the association of EMT with the particular cancer characteristic [based on by Ref. (90)].

Despite considerable experimental and computational modeling effort, the role of EMT in cancer is still not fully understood (11). In particular, the connection of EMT to various properties of cancer cells such as stemness, metabolism, and metastasis (Figure 4) is heavily debated. In many studies, EMT is considered an all-or-nothing event, whereas partial rather than full EMT may be linked to these properties (42). In this section, we will discuss modeling approaches that have been applied to obtain a better understanding of the link between EMT and cancer cell properties related to cancer progression.

### EMT and Stemness

The cancer stem cell (CSC) hypothesis states that only the small portion of tumor cells that has stem cell properties drives tumor growth in the long run because differentiated cells have a limited growth potential. The observation that immortalized human mammary epithelial cell (HMLE) populations undergoing EMT start expressing stem cell markers was reported independently by two groups in 2008 (63, 64). There are two potential explanations for these observations: first, EMT allows differentiated cells to de-differentiate into CSCs. Second, EMT leads to upregulation of self-renewal of preexisting CSCs. To distinguish between these hypotheses, Turner and Kohandel developed compartment models for both scenarios to study which best fitted the available experimental data. Because of the limited resolution and limited number of data points, Turner and Kohandel could not distinguish between the two scenarios but offered testable predictions that would distinguish the two (65).

The model by Turner and Kohandel (65) did not explicitly include EMT but merely its assumed effects (either dedifferentiation or upregulated self-renewal). By contrast, Jolly et al. attempted to elucidate the EMT-stemness interplay by combining the decision-making modules of stemness (66) and EMT (27) into a single model (67). They conclude that the position of the “stemness window” on the “EMT axis” (i.e., which EMT phenotypes allow cells to gain stemness) is not universal but can be fine-tuned by PSFs such as OVOL and GRHL2 (44). An important observation from the model is that PSFs such as OVOL can preclude association of stemness with the mesenchymal phenotype and can associate stemness exclusively to the hybrid E/M phenotype (67).

In a recent study, Sehl et al. developed a stochastic population model of the breast cancer stem cell (BCSC) niche to investigate CSC-eradication strategies for use in the clinic (68). Apart from the stem cell environment, they also took the existence of different CSC states into account, i.e., an epithelial BCSC that is proliferative and marked by ALDH expression, and a mesenchymal BCSC that is quiescent yet capable of tissue invasion and metastasis, marked by CD44+/CD24− surface marker expression. Next, they studied the effect of inhibition of various environmental EMT-inducing (e.g., IL-6 and TGFβ) and MET-inducing (e.g., BMPs, HER2, and miR-93) signals on tumor composition in the model. Inhibition of IL-6 caused an increase in the number of epithelial BCSCs because of their proliferative nature. Moreover, inhibition of BMP and miR-93 caused a decrease in total BCSCs yet also a high proportion of invasive mesenchymal BCSCs. Combinatorial treatment in the model predicted that concurrent inhibition of HER2 and IL-6 would strongly reduce the total number of BCSCs, which is in agreement with earlier experimental findings. This treatment will be further examined in a planned clinical trial (68). An interesting extension of this model would be the inclusion of a hybrid E/M BCSC phenotype given the potential association of stemness with hybrid EMT.

These studies taken together, and in particular the potential association of stemness with hybrid EMT, may explain observations of stemness not being linked to EMT (5, 69). However, it would be useful to study additional regulators in such models, which will help to elucidate the difference in tumor-initiating capacity following EMT induction by different EMT-TFs, such as SLUG and SNAIL (33), and PRRX1 (31).

### EMT and Tumor Metabolism

Another EMT-associated hallmark of cancer is altered tumor metabolism, of which the Warburg effect is the most well-known example. The increased glucose uptake of many cancer cells compared to normal cells has been extensively studied in the context of proliferation, but there is increasing evidence for its role in cancer cell migration (70) and EMT (71). Examples include lactate-induced EMT *via* TGFβ (72, 73), Twist-induced metabolic changes through the PI3K/AKT pathway (74), and involvement of key EMT suppressor miR-200 (75), either by miR-200 suppression upon the loss of fumarate hydratase (76) or by miR200c-SIRT2 regulation of metabolic reprogramming (77). These examples illustrate the complexity of the interplay between EMT and metabolic reprogramming, which is why gaining new biological insight from the vast amount of omics data is a major challenge.

Computational approaches can help in integrating these omics data to unravel the relation between EMT and metabolism. In a genome-scale metabolic model, a metabolic network is constructed of all biochemical transformations of the cell or organism of interest. This metabolic network is represented by a stoichiometric matrix, which contains the coefficients of metabolic reactions, supplemented by a mapping of reactions to the involved genes and proteins. These models can be analyzed using constraint-based modeling and flux balance analysis (78, 79).

Choudhary et al. (80) used a genome-scale metabolic model to study the metabolic cross talk as a result of dysregulation of the EGFR pathway during EMT. A stoichiometric model of the EGFR signaling network was generated from the EGFR pathway map in the Reactome database (81). To generate EMT-specific models, Choudhary and coworkers constrained the stoichiometric model with microarray gene expression data of the human breast epithelial cell line D492 and its mesenchymal-like counterpart D492M, resulting in a separate epithelial and mesenchymal model. Flux balance analysis predicted decreased signaling in the AKT pathway in the mesenchymal cell line, and since AKT activation increases glucose uptake and glycolysis (74), this suggested an EMT-related decrease in glycolysis. Measurements of glucose uptake and lactate secretion *in vitro* confirmed this decrease in glycolysis (80). In addition, they identified genes responsible for EMT suppression or reversion in D492 cells by studying how the flux through the “mesenchymal” model could be made more similar to the flux in the “epithelial” model, illustrating the model’s usefulness in understanding both cancer metabolism and EMT, and their combination (80).

Interestingly, these results are cell line specific: constraining the stoichiometric model with gene expression data from HMLE, MCF7, and MCF10A cells and their mesenchymal counterparts (i.e., after EMT induction), and repeating the flux-based analysis predicted that mesenchymal HMLE cells had less signaling in the AKT pathway compared to their epithelial counterpart, such as the D492 cells. However, the mesenchymal MCF7 and MCF10A cells had increased AKT signaling, which is in agreement with the increased glucose uptake and glycolysis reported in earlier studies (74, 82).

### EMT and Migration, Invasion, and Metastasis

Enhanced migratory and invasive capacities are well-known properties of mesenchymal cells and therefore a key aspect of EMT. By secreting matrix metalloproteinases (MMPs), mesenchymal cells remodel and degrade the ECM and migrate as “path generators.” By contrast, amoeboid cells, another individually migrating phenotype, do not secrete MMPs but have a high morphological plasticity, allowing them to squeeze into ECM gaps, acting as “path finders” (83). Tumor cells can switch between these phenotypes in a process known as the amoeboid–mesenchymal transition (AMT) and its reverse mesenchymal–amoeboid transition (MAT). First, Huang et al. identified and modeled the GTPase-based Rac/Rho circuit as a three-way switch of AMT, allowing for partial AMT with a hybrid A/M phenotype (83). Next, they combined this AMT decision model with their previously developed EMT decision model (27) to offer a more detailed understanding of the observed migratory phenotypes and the transitions between them (84). One possible transition concerns the collective (hybrid E/M) to amoeboid transition (CAT) and its reverse ACT, observed in melanoma (85) and fibrosarcoma (86) cells. In general, the five phenotypes (E, E/M, A, A/M, and M) and the three types of transitions between them (EMT/MET, AMT/MAT, and CAT/ACT) illustrate the rich plasticity in migratory phenotypes tumor cells can employ during metastasis (84). For a better understanding of the dynamics of these transitions, specifically the spatiotemporal interplay of the proposed regulatory model with both cell shape and migration, the model could be combined with the model by Holmes et al. (87), who showed how the combination of intracellular Rac/Rho and ECM signaling regulates lamellipodial dynamics.

Although the increased migratory and invasive capacity of the mesenchymal phenotype suggest a role for EMT in metastasis (2), the prerequisites for metastasis are not yet fully understood regarding the role of full or partial EMT (11). This lack of understanding limits the applicability of computational modeling to the use of phenomenological models. One such model was developed by Cohen et al. (88), who constructed an extensive, literature-derived Boolean model to predict metastasis. The output of this model consists of the broad, descriptive phenomena “Cell Cycle Arrest,” “Apoptosis,” and “Metastasis.” “Metastasis” was considered to depend on the states of “EMT,” “Invasion,” and “Migration,” each of which connects to the gene regulatory network part of the model.

Using gain-of-function (GoF) and loss-of-function (LoF) perturbations (forcing genes in the model to be either ON or OFF), the model could reproduce the phenotypic results of various published mutations (88). Additionally, Cohen and coworkers explored the model by testing single and double permutations and their influence on the occurrence of metastasis. One double mutation that always resulted in metastasis in the model was the synergistic notch intracellular domain GoF and p53 LoF (88), which indeed led to metastasis in experimental work (89). An unanswered question is how the results of the model depend on the assumption that EMT is required for metastasis, as that may be context dependent (6–10). Therefore, the true role of EMT in metastasis remains a topic for further research, especially because some parts of the metastatic cascade, such as intravasation, have received little attention (11).

### EMT and Tumor Cell Interactions

Because pathogenic EMT, and cancer in general, can be viewed as a disease of tissue development, we must ultimately also take into account cellular interactions and interactions with the environment (90, 91). This can be done by employing hybrid or multiscale models that include both cell-autonomous processes (often an ODE or stochastic model) and spatial interactions [for a general review of spatial models, see Ref. (92)]. These models can be useful to help understand spatial aspects of EMT, such as collective migration or tumor budding (the invasion of malignant cells into the supporting stroma) (93), and spatiotemporal regulation of EMT taking into account different conditions in the primary tumor and at distant sites (94).

An example of a spatial model of EMT, albeit not on its role in cancer but its role in cardiac cushion formation during heart development (95) employed the Cellular Potts Model. This is a lattice-based model where each cell is represented by a collection of lattice sites (96, 97). This type of model is driven by differential adhesion properties between the epithelial and mesenchymal cells and the ECM, i.e., the fibrous structures within tissues that provide support to cells and that contain various growth factors. EMT was simulated by each cell having a constant probability of transitioning from the epithelial to the mesenchymal phenotype. Despite the simplicity of the model, it was able to produce structures resembling cardiac cushions.

Other studies have looked into the expected impact of signaling processes between neighboring cells on pattern formation. One example of such a multiscale model is the individual-based model by Ramis-Conde et al. (98), who showed how E-cadherin–β-catenin dynamics could lead to spatial configuration changes in epithelial layers. In their model, reorganization is driven by free β-catenin levels in the nucleus: a cell with high nuclear free β-catenin is prone to start migrating, after which it exerts physical forces on the E-cadherin–β-catenin bonds with neighboring cells. When these bonds break, the β-catenin in the neighboring cells used in the intercellular bonds becomes free β-catenin and translocates to the nucleus. Repetition of this process sends a “wave” of detachment through the epithelial cell layer (98).

Another example of a multiscale model of EMT is the extension of the TCS model with Notch signaling by Boareto et al. (99). Notch signaling can cause neighboring cells to adopt a different fate (when the Notch receptor receives signals from the Delta ligand expressed in neighboring cells) or a similar fate (when the Notch receptor receives signals from the Jagged ligand). Because the Notch and EMT circuits are highly connected, Notch signaling can cause neighboring cells to adopt a different or similar EMT phenotype. Boareto and coworkers employed a hexagonal-lattice model in which each lattice site is a single cell and showed that Notch-Jagged signaling could act as a PSF because it induces neighbors to adopt the same phenotype. They showed that both types of Notch signaling could induce EMT, yet Notch-Jagged signaling allows for the formation of clusters of hybrid E/M cells. Because these CTC clusters have a high metastatic potential (15), this work predicts that Jagged might be an interesting therapeutic target to mitigate this potential (99). However, it is unknown if Notch-Jagged signaling indeed has a role in the formation and maintenance of CTC clusters.

Spatial simulations can also be employed at the scale of an entire tumor. Such an approach was employed by Waclaw et al. (100) to investigate how tumor cell dispersal assisted by EMT and cell turnover affect lesion growth and regrowth after targeted therapy. The cells in this model replicated stochastically with a probability proportional to the empty space around them. After targeted therapy, which causes sensitive cells in the lesion to die, the resistant cells could disperse to the area previously occupied by the sensitive cells, thereby accelerating regrowth of the lesion (100). Although EMT was included in the model only implicitly as a source for the short-range dispersal, these simulations suggest that EMT may play a role not only in metastasis but also in tumor growth and regrowth.

In summary, these recent computational EMT models show how including spatial aspects into simulations helps in the understanding of tumor progression. In most cases, the spatial dimension was limited to cell–cell interactions within a “monoculture” of tumor cells. An open question is how tumor cells interact with other recruited cells within the tumor microenvironment (90). These interactions are critical in invasion into the ECM and immune cell activity. For example, macrophages can take up half the mass of breast tumors, and their presence at the tumor site correlates with poor prognosis (101). Knútsdóttir et al. (102, 103) used a three-dimensional individual cell-based model to show how EGF/CSF-1 paracrine signaling causes tumor cells and macrophages to comigrate (103), which leads to macrophage-assisted invasion and intravasation (104). Because CCL18 from tumor-associated macrophages (TAMs) can induce EMT in breast cancer cells, and mesenchymal breast cancer cells can activate macrophages to a TAM-like phenotype by CSF2 (105), it would be important to include the effect of EMT on the macrophage-assisted migration of tumor cells within similar spatial models.

## Perspective

Epithelial–mesenchymal transition has been implicated to play a role in various changes in tumor characteristics, and some of these changes have received very little modeling attention (Figure 4). One of these is the evasion of immune destruction, which is considered a hallmark of cancer (90). An indication that this hallmark is related to EMT is the role of TGFβ, which is not only a potent EMT inducer but also a critical regulator of T-cell development and homeostasis (106). Recent reports have strengthened this relation by showing that hypoxia-induced EMT is linked to immune resistance (107), and that miR-200 and ZEB1, both part of the EMT core regulatory network, are involved in upregulation of the immune checkpoint programmed death ligand-1 (108). Apart from Tripathi et al. (109), who developed an ODE model to study the involvement of miR-200 in immunoproteasome regulation, the relation between EMT and immune evasion has received no modeling attention. Future computational modeling of this relation might prove useful to elucidate underlying mechanisms, e.g., with respect to optimization of immune checkpoint therapies. Another EMT-related tumor characteristic is resistance to chemotherapy. Radiation and chemotherapy can cause a phenotypic transition of cells to a resistant state (110, 111) while undergoing a partial EMT (42). Moreover, suppression of EMT in pancreatic (6) and lung cancer (7) (re)sensitizes cells to chemotherapy. Unraveling the underlying mechanisms of EMT-related chemoresistance awaits future experimental and computational studies.

Over the last decade, computational biology has become increasingly recognized as a valuable tool in the biologists’ toolbox. As discussed in this review, its interplay with experimental biology has advanced our understanding of the role of EMT in tumor progression in a synergistic fashion. Given the ongoing increase in publically available, high-quality experimental data sets as well as in computational tools, such multidisciplinary approaches are key to achieve a quantitative understanding of cancer biology. Thus, as foreseen by Hanahan and Weinberg (48), cancer research will indeed become a logical science, in which the complexities of the disease, such as the role of EMT, are understood in terms of their underlying principles.

## Author Contributions

GB and JB conceived the manuscript and its structure; GB drafted the manuscript and composed the figures; ED and JB critically revised the manuscript. All authors read and approved the final manuscript.

## Conflict of Interest Statement

The authors declare that the research was conducted in the absence of any commercial or financial relationships that could be construed as a potential conflict of interest.

## Abbreviations

ACT, amoeboid collective transition; AMT, amoeboid–mesenchymal transition; BCSC, breast cancer stem cell; CAT, collective amoeboid transition; CBS model, cascading bistable switches model; CFL, coupled feedback loop; CSC, cancer stem cell; CTC, circulating tumor cell; E/M, epithelial/mesenchymal (hybrid); ECM, extracellular matrix; EMT, epithelial mesenchymal transition; GoF, gain-of-function; HCC, hepatocellular carcinoma; LoF, loss-of-function; MAT, mesenchymal–amoeboid transition; MET, mesenchymal–epithelial transition; MMPs, matrix metalloproteinases; ODE, ordinary differential equation; POET, Pareto optimal ensemble technique; PSF, phenotypic stability factor; TAM, tumor-associated macrophage; TCS model, ternary chimera switch model; TF, transcription factor.
